# Spatio-Temporal Heterogeneity of Ecological Quality in a Typical Dryland of Northern China Driven by Climate Change and Human Activities

**DOI:** 10.3390/plants13233341

**Published:** 2024-11-28

**Authors:** Shuai Li, Junliang Gao, Pu Guo, Ge Zhang, Yu Ren, Qi Lu, Qinwen Bai, Jiahua Lu

**Affiliations:** 1 Institute of Desertification Studies, Institute of Ecological Conservation and Restoration, Chinese Academy of Forestry, Beijing 100091, China; lisshaxs@163.com; 2 Inner Mongolia Dengkou Desert Ecosystem National Observation Research Station, Experimental Center of Desert Forestry, Chinese Academy of Forestry, Dengkou 015200, China; gaojl@caf.ac.cn (J.G.); tozhangge@163.com (G.Z.); bqw1686330558@163.com (Q.B.); ljh15147125358@163.com (J.L.); 3 National Natural History Museum of China, Beijing 100050, China; guopu@caf.ac.cn; 4 Ordos Vocational College of Ecological Environment, Ordos 017010, China; nmryu@126.com

**Keywords:** remote sensing ecological index, ecological quality, dryland, spatio-temporal change, hurst exponent, multiple residual regression analysis

## Abstract

With the intensification of climate change and anthropogenic impacts, the ecological environment in drylands faces serious challenges, underscoring the necessity for regionally adapted ecological quality evaluation. This study evaluates the suitability of the original Remote Sensing Ecological Index (oRSEI), modified RSEI (mRSEI), and adapted RSEI (aRSEI) in a typical dryland region of northern China. Spatio-temporal changes in ecological quality from 2000 to 2022 were analyzed using Theil–Sen median trend analysis, the Mann–Kendall test, and the Hurst exponent. Multiple regression residual analysis quantified the relative contributions of climate change and human activities to ecological quality changes. Results showed that (1) the aRSEI was the most suitable index for the study area; (2) observed changes exhibited significant spatial heterogeneity, with improvements generally in the inner areas of the Yellow River and declines in the outer areas; and (3) changes in ecological quality were primarily driven by climate change and human activities, with human activities dominating from 2000 to 2011 and the influence of climate change increasing from 2012 to 2022. This study compares the efficacy of RSEIs in evaluating dryland ecological quality, identifies spatio-temporal change patterns, and elucidates driving mechanisms, offering scientific evidence and policy recommendations for targeted conservation and restoration measures to address future changes in dryland regions.

## 1. Introduction

The rapid expansion of the global population and economy has significantly under-mined the stability and service of ecosystems [1,2], posing a severe threat to biodiversity [3]. In the face of climate change, dryland ecosystems are particularly vulnerable to these challenges [4]. Therefore, it is crucial to conduct regionally adapted evaluations of ecological conditions in drylands. Such evaluations not only facilitate an understanding of the current status of dryland ecosystems but also provide a scientific foundation for regional environmental protection and ecological restoration [5].

Remote sensing technology offers a number of advantages, including the capacity to provide extensive coverage, rapid data acquisition, and reduced cost [6]. It provides multi-temporal and multi-scale ecological environment information and has become an important method of ecological condition evaluation. The Remote Sensing Ecological Index (RSEI) developed by Xu et al. [7] is the most commonly used index for evaluating ecological conditions in terms of quality. The RSEI is based on four ecological indicators: greenness, humidity, dryness, and heat. This index has numerous advantages and has been extensively employed for evaluating ecological quality at diverse scales and in different regions [8,9,10,11]. Notably, in various regional research studies, some scholars have proposed modifications to the original RSEI (oRSEI), which was constructed using the Normalized Difference Vegetation Index (NDVI), Moisture Index (WET), Normalized Difference Built-Up Index (NDBSI), and Land Surface Temperature (LST), to enhance its applicability in specific regions [12,13,14,15]. For example, Liu et al. [14] incorporated the Aerosol Optical Depth (AOD) into the oRSEI to evaluate the ecological environment in Beijing. Similarly, Gao et al. employed the Biophysical Composition Index (BCI) in place of the NDBSI to evaluate the urban ecological quality in Lanzhou, China [16]. Yang et al. added the Vegetative Health Index (VHI) to evaluate the ecological quality in the Guangdong–Hong Kong–Macau Greater Bay Area of China [17]. Furthermore, some studies have devised novel RSEIs by selecting ecological indicators that exhibit high correlation with the oRSEI [18,19]. Modifications of the RSEI in drylands are typically made with a salinity indicator [20,21], which enhances the index’s ability to reflect the specific characteristics of dryland ecosystems.

Nevertheless, these studies have not yet conducted a comprehensive assessment of the advantages and disadvantages of the modified index from multiple perspectives. To address this gap, this study selected a typical dryland area in northern China as a case study. It assessed the suitability of three indices: the oRSEI, the modified RSEI (mRSEI) with an added salinity index (ST), and the adapted RSEI (aRSEI) which replaced the NDVI with the Modified Soil-Adjusted Vegetation Index (MSAVI).

The study area is located in the “U-shaped” region of the Yellow River Basin, a significant region of concern for desertification control in China. It is also a critical region for biodiversity conservation and the implementation of the national strategy for ecological protection and high-quality development of the Yellow River Basin [22]. This region encompasses a diverse range of climatic zones and landscapes, making it highly representative of global drylands. Various environmental restoration initiatives have been implemented here, such as the Three-North Shelterbelt Program, the Returning Farmland to Forest Program, and the Returning Grazing Land to Grassland Program [22,23]. These projects have significantly altered the region’s ecological status. A regionally adapted evaluation of changes in ecological quality will provide a scientific basis and reference for ecological management in the region.

Through this study, we aim to (1) assess the efficacy of three remote sensing ecological indices in a typical dryland; (2) utilize the optimal index to reveal the spatial and temporal changes in ecological quality in the study area during 2000–2022; (3) quantitatively evaluate the impacts of climate change and human activities on ecological quality changes through multiple regression residual analysis; and (4) conduct combinations with persistence analysis to propose regional ecological protection and management strategies. These efforts will simultaneously provide theoretical support for ecological protection and restoration in both China and the global drylands.

## 2. Results

### 2.1. Comparison of RSEIs

#### 2.1.1. PCA and Correlation Analysis

The PCA results of the oRSEI, aRSEI, and mRSEI (Table 1) showed that the contribution of the PC1 was more than 82%, indicating that the PC1 effectively synthesizes the characteristic information of the ecological indices. After the “1-PC1” operation, greenness and humidity positively affected ecological quality, while the heat, dryness, and salinity contributed negatively.

The correlation analysis (Table 2) demonstrated that the three RSEIs had a strong correlation with the selected ecological indicators. The absolute values of the correlations with each ecological indicator ranged from 0.8481 to 0.9378, indicating that all three RSEIs effectively characterized the regional ecological quality status. Nevertheless, notable differences were observed among the specific indices. The oRSEI showed the lowest mean value of correlation with each indicator (0.9049), having the highest correlation with the NDVI and NDBSI; the lowest correlation with the MSAVI, WET, and SIT; and an LST within the range of the aRSEI and mRSEI. The mRSEI had the highest mean value of correlation with each indicator (0.9078), with the highest correlation observed with the MSAVI, WET, and the ST. However, it had the lowest correlation with LST and the NDBSI. The aRSEI exhibited a mean correlation with indicators that is intermediate between the aRSEI and mRSEI, with the highest correlation with LST and the lowest correlation with the NDVI. The correlations with other indicators were all intermediate between the oRSEI and mRSEI. This indicates a certain balance in the evaluation of ecological quality across various ecological indicators.

#### 2.1.2. Spatial Differences

**Figure 1:**
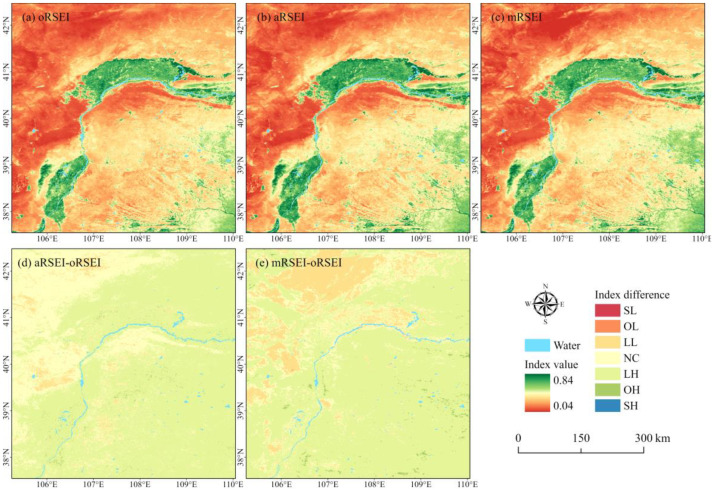
Spatial distribution of and differences in three indices in 2022. (**a**–**c**): Spatial distribution of the oRSEI, aRSEI, and mRSEI, respectively. (**d**) Spatial differences between the oRSEI and aRSEI. (**e**) Spatial differences between the oRSEI and mRSEI. Note: SL: significantly low; OL: obviously low; LL: slightly low; NC: no change; LH: slightly high; OH: obviously high; SH: significantly high.

The results of the oRSEI, aRSEI, and mRSEI exhibited the same spatial distribution pattern (Figure 1a–c). All indices showed good ecological quality in forests and croplands, which was relatively low in steppes, and the lowest quality was observed in deserts and the Gobi, which have unvegetated areas and extremely low vegetation cover. However, the differences between the aRSEI and oRSEI (Figure 1d) indicated that the aRSEI slightly increased the value of most vegetated areas while noticeably decreasing the quality of unvegetated areas, with essentially no effect on areas of extremely low vegetation. In contrast, the mRSEI showed a larger difference compared to the oRSEI (Figure 1e). It obviously decreased the value in areas with relatively high salinity content, while slightly increasing the value in unvegetated, extremely low, low, and high vegetation cover areas. Additionally, it significantly increased the value in some mining and vegetation restoration areas.

#### 2.1.3. Time Series Differences

**Figure 2:**
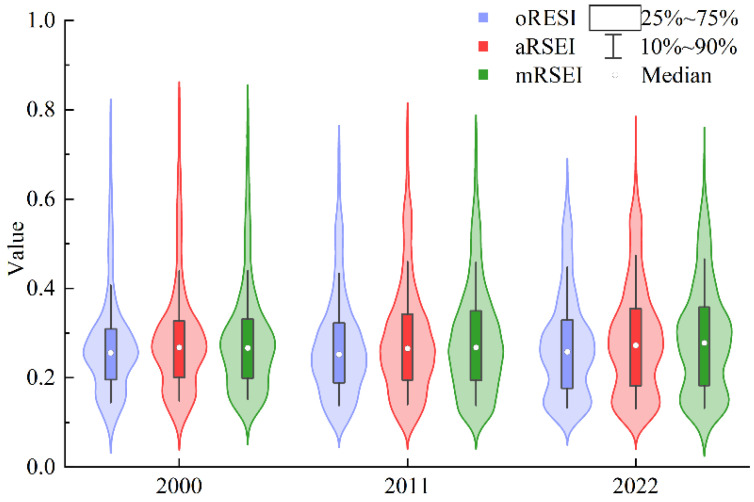
Violin plots of oRSEI, aRSEI, and mRSEI from 2000 to 2022.

Years 2000, 2011, and 2022 were selected to compare changes over time for these three RSEIs. The violin plots (Figure 2) show that these indices had similar distribution patterns and change characteristics both within individual years and over the time series. In terms of the interquartile range, the oRSEI had the least dispersion, while the ecological quality values for the aRSEI and mRSEI were more dispersed. This increased dispersion indicates a greater sensitivity to spatial heterogeneity, suggesting that the aRSEI and mRSEI are more effective for capturing and characterizing the spatial differences in ecological quality within heterogeneous regions compared to the oRSEI. In contrast, the lower dispersion of the oRSEI in heterogeneous regions may indicate a lack of sensitivity to spatial differences in ecological quality, potentially limiting its effectiveness in capturing the spatial patterns and variations within these regions.

The results of the PC1 contribution, ecological indicator correlation, spatial differences, and time series differences were combined to compare the usability of the three RSEIs. The findings indicated that all three indices had comparable applications. However, the mRSEI did not exhibit a distinct advantage over the oRSEI and may have amplified the impact of salinity content. In contrast, the aRSEI, with the NDVI substituted by the MSAVI, was more balanced overall and more accurately reflected the actual ecological conditions.

### 2.2. Spatio-Temporal Changes in Ecological Quality

#### 2.2.1. Spatial Pattern of Ecological Quality

**Figure 3:**
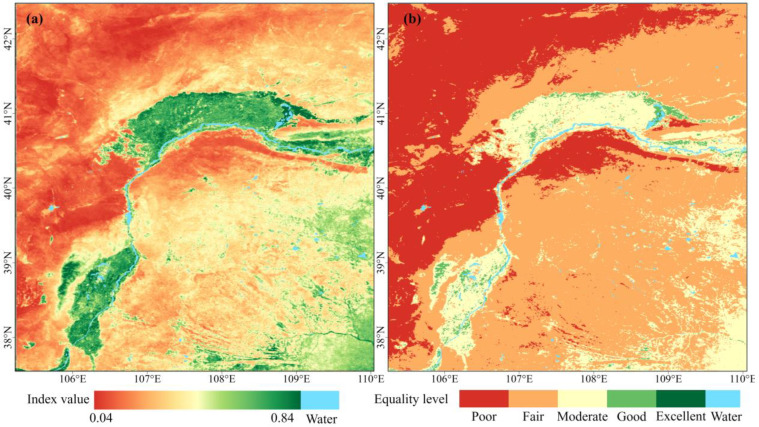
The distribution (**a**) and classification (**b**) of ecological quality in the study area in 2022.

The aRSEI was used to investigate the spatial and temporal changes in ecological quality in the study area from 2000 to 2022. Figure 3a shows the spatial pattern results of ecological quality in 2022. The aRSEI values range from 0.04 to 0.84, with obvious spatial heterogeneity. High-value areas are mainly distributed in the eastern regions of the Helan and Yin Mountains, as well as in agricultural plains such as the Yinchuan Plain and Hetao Plain, indicating the better ecological quality of these areas. Low-value areas are primarily found in the Tengger Desert, Ulan Buh Desert, and Gobi areas west of the Yin Mountains, indicating relatively poor ecological quality in these locations. The results are consistent with the actual conditions and fully reflect the differences in ecological quality across various geographic regions influenced by natural and human activities.

**Figure 4:**
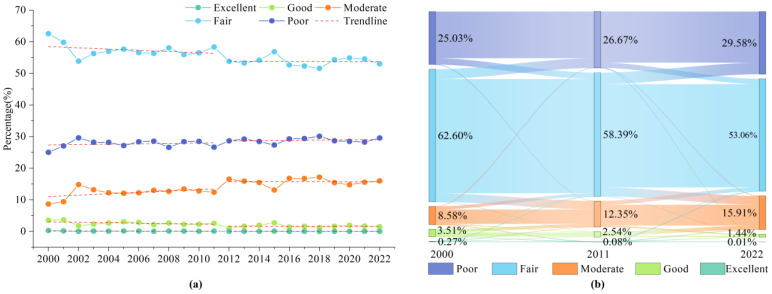
Changes in (**a**) and area transition matrix of (**b**) ecological quality levels from 2000 to 2022.

Referring to existing studies [24], the aRSEI values were classified into five classes at 0.2 intervals: Poor (0–0.2), Fair (0.2–0.4), Moderate (0.4–0.6), Good (0.6–0.8), and Excellent (0.8–1). The spatial distribution and percentages of these categories for the year 2022 are shown in Figure 3b and Figure 4b. The ecological quality was predominantly categorized as Poor and Fair, with area shares of 29.58% and 53.06%, respectively. Poor areas were mainly distributed in deserts, the Gobi, barren areas, and areas with extremely low vegetation cover. Fair areas were primarily found in scattered steppes or shrub steppes with partially low to medium vegetation cover. Moderate areas, comprising 15.91% of the total, were primarily situated in the Yinchuan Plain, Hetao Plain, and croplands in northern Ordos and Yulin. These areas also included mountainous regions with high vegetation cover, such as the Helan Mountains and the eastern Yin Mountains. Good areas, with a relatively small proportion of 1.44%, were mainly distributed in the Helan Mountains and some croplands. The Excellent areas, representing the smallest proportion (0.01%), were located in the wetland of Wuliangsu lake.

#### 2.2.2. Changes in Ecological Quality Level

Figure 4a illustrates the changes in ecological quality levels within the study area from 2000 to 2022. Overall, the proportion of the area classified as Poor demonstrated an increasing trend throughout the study period, while the Fair level exhibited a decreasing trend. The Moderate category showed an upward trend, while both Good and Excellent categories displayed a decreasing trend. In terms of temporal dynamics, the changes in ecological quality can be divided into two distinct periods, with 2011 representing a pivotal turning point.

The first period, from 2000 to 2011, is characterized by relatively drastic changes. The proportion of Poor increased slowly, while Fair decreased significantly. The Moderate category showed a substantial increase, while Good and Excellent experienced a slight decrease. The second period, from 2012 to 2022, exhibits more gradual changes. Poor increased more slowly, while Fair and Moderate categories decreased gradually, and Good and Excellent categories also showed a more gradual decrease.

To gain deeper insights into these two periods, we conducted a class transfer analysis, the results of which are presented in Figure 4b. From 2000 to 2011, the increase in Poor classifications was primarily due to the downgrading of Fair areas. While some Fair areas were also upgraded to Moderate, the overall trend still resulted in an increase in Poor classifications. The Fair category experienced a 4.21% decrease in area share, while the Moderate category saw a 3.77% increase. Good and Excellent categories were largely downgraded to Moderate and Good categories, respectively, with their area shares decreasing by 0.97% and 0.19%.

During the 2011–2022 period, the direction of transfers between levels remained consistent with that of the previous period. However, except for the Poor category, the rate of transfer was significantly lower than that in 2000–2011. It is notable that a very small percentage of the area in both time periods underwent large-scale transfers across multiple classes. These findings not only reveal the differentiation of ecological quality in the study area across different periods but also reflect the difficulty of directly transitioning from Poor or Fair to Good or Excellent categories in drylands with challenging ecological environments.

#### 2.2.3. Spatio-Temporal Trends of Ecological Quality

**Figure 5:**
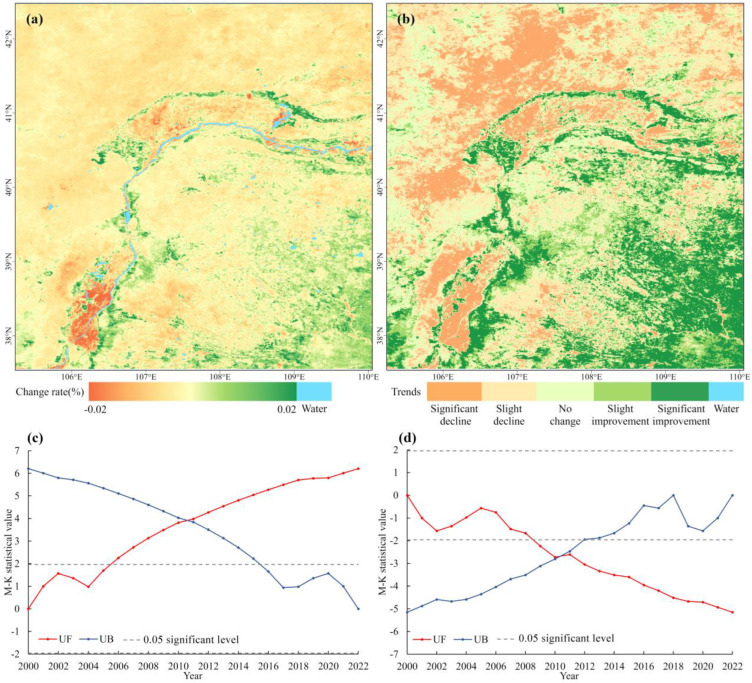
Time series trend of ecological quality, Mann–Kendall significance test, and Mann–Kendall mutation test in the significant-change area. (**a**) Theil–Sen median trend analysis from 2000 to 2022. (**b**) Theil–Sen median trend analysis and Mann–Kendall significant test from 2000 to 2022. (**c**) Mann–Kendall mutation test in the significant-improvement areas. (**d**) Mann–Kendall mutation test in the significant-decline areas. Note: UF: forward trend statistical value; UB: backward trend statistical value.

Figure 5 presents the results of ecological quality change trends in the study area from 2000 to 2022, based on Theil–Sen median trend analysis and the Mann–Kendall test. The rate of change in ecological quality ranged from −0.02 to 0.02 per year, showing an overall normal distribution. Areas showing an increasing trend in the aRSEI were mainly distributed in the inner areas (east and south) of the Yellow River and around the Yinchuan Plain and Hetao Plain, while areas with a decreasing trend were mainly found in the outer areas (west and north) of the Yellow River and within the Yinchuan Plain and Hetao Plain. Based on the significance test results, the study area was categorized into five change classifications: significant improvement, slight improvement, no change, slight decline, and significant decline (Figure 5b). The results demonstrated that 30.72% of the region exhibited an improvement, 46.45% experienced a decline, and 22.83% remained unchanged during the study period.

A clear spatial heterogeneity was observed between areas of improvement and decline, with most of the former located in the inner areas (east and south) of the Yellow River, and most of the decline in the outer areas (west and north) of the Yellow River. This polarized change is also evident in the kernel density distribution of ecological quality, as shown in Table 3. Areas with significant changes in ecological quality account for 34.86% of the study area. Areas with significant improvement account for 16.77%, mainly concentrated in the eastern foothills of Helan Mountains, the Yellow River coast, the northeastern part of the Ulan Buh Desert, the southern foothills of the Yin Mountains, most of the Mu Us Sandy Land, and the eastern part of the Ordos Steppe. Areas with significant decline account for 18.09% and are distributed across the Yinchuan Plain, the Hetao Plain, the Helan Mountains, the central part of the Ulan Buh Desert, parts of the Yin Mountains, and Gobi regions situated to the west of the Yin Mountains.

To ascertain the timing of significant differences and whether any mutations occurred, we conducted an M-K mutation test on the means of the significant-change areas from 2000 to 2022 (Figure 5c,d). The UF curves for the significant-improvement areas indicate that the ecological quality of this region improved over the 23-year period, reaching a level of significant difference in 2006. The UF curves for the significant-decline area show a decline in ecological quality, reaching a level of significant difference in 2009. Notably, both the UF and UB curves intersected outside the 0.05 confidence interval around 2011, indicating substantial changes in both the significant-improvement and -decline areas around this time. This finding corroborates the significant shift in the percentage distribution of ecological quality levels before and after 2011, as previously shown.

### 2.3. Persistence and Future Trends

**Figure 6:**
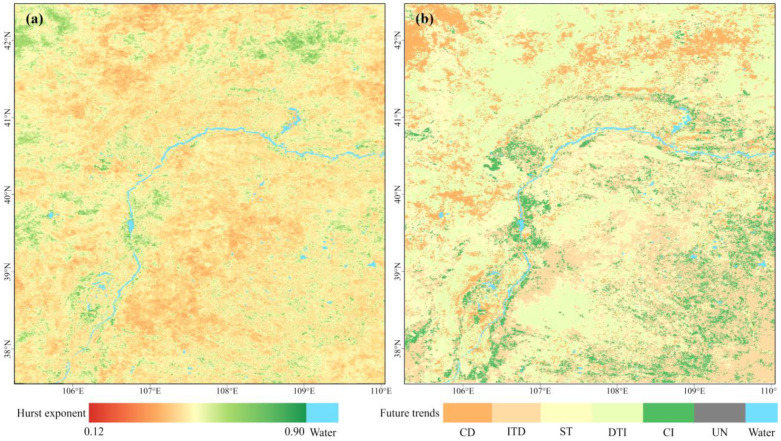
Hurst exponent (**a**) and future trends (**b**) in the study area. Note: CD: continuous decline, ITD: improvement to decline, ST: stable, DTI: decline to improvement, CI: continuous improvement, UN: uncertain.

To investigate the persistence of existing change trends, the Hurst exponent was calculated. The values ranged from 0.1 to 0.9, with a mean value of 0.45 (Figure 6a). Areas likely to exhibit positive persistent change in ecological quality in the future (Hurst > 0.5) accounted for 42.4% of the study area. These areas were primarily located in the Yinchuan plain, Ulan Buh Desert, Gobi Desert, Ulat-Damao Steppe, Mu Us Sandy Land, and eastern part of the Ordos Steppe. Conversely, areas prone to anti-sustained change (Hurst < 0.5) comprised 56.95% of the study area, with a concentration in the western part of the Mu Us Sandy Land and Ordos Steppe, as well as in the deserts and Gobi regions.

Furthermore, the persistence of ecological quality changes was categorized and assessed by combining the Hurst exponent with Theil–Sen median trend analysis (Figure 6b and Table 4). The results indicated that 8.75% of the region is likely to experience continuous improvement in the future, 11.76% may continuously decline, and 21.89% is expected to remain stable. In 56.95% of the region, the existing trends appear to be unstable. Among these regions, 34.87% may transition from decline to improvement, while 22.08% might shift from improvement to decline. For 0.65% of the region, future trends are uncertain.

### 2.4. Effects of Climate and Human Activities on Ecological Quality

#### 2.4.1. Effects Analysis for the Period 2000–2022

**Figure 7:**
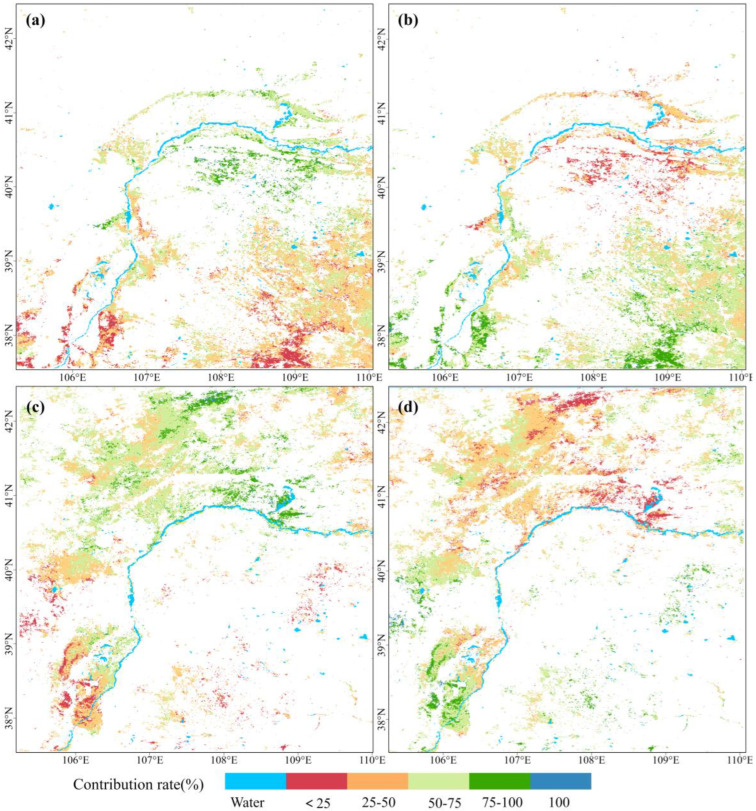
Spatial distribution of climate change and human activity contributions to ecological quality change in significant-change areas. (**a**) Climate change contribution in the significant-improvement areas. (**b**) Human activity contribution in the significant-improvement areas. (**c**) Climate change contribution in the significant-decline areas. (**d**) Human activity contribution in the significant-decline areas.

**Figure 8:**
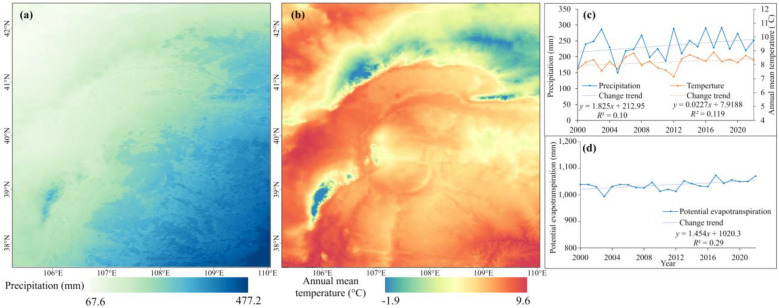
Spatial pattern of and changes in climate factors. (**a**) Spatial pattern of annual precipitation. (**b**) Spatial pattern of annual mean temperature. (**c**,**d**) Changes in annual precipitation, annual mean temperature, and potential evapotranspiration.

The areas with significant changes during 2000–2022 were selected to quantitatively analyze the effects of climate change and human activities on ecological quality changes. The results are presented in Table 5 and Figure 7. The spatial pattern of and changes in climate factors are shown in Figure 8.

In significant-improvement areas, only 0.69% was solely driven by climate change, sporadically distributed in the Kubuqi Desert. Areas solely driven by human activities accounted for 0.53% of the total, located in the southeast edge of the Mu Us Sandy Land and the east and west foothills of the southern Helan Mountains. The remaining areas experienced changes driven by both factors. Climate change was the primary driver (contribution rate > 50%) in 38.89% of the area, concentrated in the Kubuqi Desert, along the Yellow River, the southern foothills of the Yin Mountains, and the northeastern part of the Ulan Buh Desert. Human activities were the primary driving force in 59.94% of the area, particularly in the eastern and western foothills of the Helan Mountains, the Yinchuan Plain to the east of the Yellow River, and much of the Mu Us Sandy Land. Overall, in areas where ecological quality significantly improved, climate change and human activities contributed 46.32% and 53.68%, respectively.

In significant-decline areas, changes were also primarily driven by a combination of climate change and human activities, but with different patterns. Climate change was the main driver (contribution rate > 50%) in 57.44% of the area, primarily in the northern part of the Yinchuan Plain and Helan Mountains, the central and eastern parts of the Hetao Plain, Yin Mountains, and the desert and Gobi areas. In 41.05% of the area, human activities were identified as the primary driver, mainly in the southern part of the Yinchuan Plain and Helan Mountains; the western part of the Ulan Buh Desert; and localized areas in the Mu Us Sandy Land, Ordos Steppe, and Ulat-Damao Steppe. The contributions of climate change and human activities in significantly declining areas were 52.69% and 47.31%, respectively.

Overall, the drivers of ecological quality changes show clear spatial heterogeneity Essentially demarcated by the southern edge of the Kubuqi Desert, changes in the southern parts are primarily driven by human activities, while those in the northern parts are mainly driven by climate change. The contributions of climate change and human activities to changes in areas of significant change are relatively balanced, with human activities contributing slightly more in areas of significant improvement and climate change contributing slightly more in areas of significant decline.

#### 2.4.2. Effects Analysis by Periods

In order to identify differences in driving forces between the two periods, a separate analysis was conducted for each period of change in the study area.

As shown in Table 5, during 2000–2011, areas driven solely by climate change accounted for 8.07% of the significant-improvement area and 6.12% of the significant-decline area. Areas driven solely by human activities reached 20.13% and 52.94%, respectively. In areas where both climate change and human activities exerted influence, climate change was the predominant driver (contribution rate > 50%) in 14.75% of improved areas and 6.2% of declined areas. Conversely, human activities were the primary driver in 57.06% and 34.75% of these areas, respectively. The overall contributions of climate change and human activities during 2000–2011 were 31.69% and 68.31% for significant-improvement areas, and 16.64% and 83.36% for significant-decline areas, respectively.

During 2012–2022, the influence of climate change and human activities shifted significantly. In the significant-improvement area, the proportion of areas solely and predominantly driven by climate change increased to 19.10% and 44.60%, while areas driven by human activities decreased to 10.74% and 24.77%, respectively. Similar changes were observed in the significant-decline area, with areas solely and predominantly driven by climate change increasing to 17.18% and 31.66%, while areas driven by human activities decreased to 17.75% and 33.42%, respectively. For this period, the contributions of climate change and human activities were 59.67% and 40.33% in significant-improvement areas, and 49.03% and 50.97% in significant-decline areas, respectively.

In conclusion, human activities were the primary driver of ecological quality changes in the study area from 2000 to 2011. In contrast, the dominant drivers underwent a significant shift between 2012 and 2022, with climate change playing a substantially stronger role and human activities having a relatively weaker influence.

## 3. Discussion

### 3.1. Regional Modifications of RSEI

The construction of the RSEI by Xu et al. [7] reduces the influence of single indicators and subjective factors, simplifies data collection, and enables quantitative analysis and visualization of ecological quality based on long time series data [25,26]. These advantages have made the RSEI a widely used methodology in ecological quality evaluation [27]. Nevertheless, in order to enhance its applicability in specific regions, numerous studies have modified or reconstructed the oRSEI [28,29].

In this study, we introduced a salinity index and constructed an mRSEI, comparing its applicability with the oRSEI in a typical dryland of northern China. The results demonstrated that both the oRSEI and mRSEI effectively characterize regional ecological quality and accurately reflect spatial variations, with evaluations based on the contribution rate of the PC1, correlation with ecological indicators, spatial differences, and temporal variations. In areas with higher salinity, ecosystems are more fragile due to the influence of salt. The incorporation of the salinity index allows for a more comprehensive reflection of the negative impact of salinity on ecosystems, resulting in a clear decline in the ecological quality value in high-salinity areas. Conversely, in areas with lower salinity, where the impact of salinity on ecosystem quality is minimal, the addition of the salinity index results in a perceived improvement in ecological quality. This does not necessarily reflect the actual ecological conditions but rather an increased sensitivity of the mRSEI to salinity, which has the potential to mask the true ecological quality in low-salinity areas and potentially distort the evaluation results. While there was minimal overall discrepancy between the values of the two indices, the introduction of the salinity index resulted in a notable divergence in ecological quality values. This indicates potential inconsistencies when modifying the oRSEI with additional ecological indicators.

Furthermore, we examined several case studies in which the oRSEI was adapted on a regional basis by integrating additional ecological indicators. For example, Wang J et al. added a salinity index to evaluate the ecological quality in the Ulan Buh Desert, finding minimal differences between the modified RSEI and oRSEI except in areas heavily affected by salinity [20]. Zhang et al. incorporated salinity and water network density indices in drylands, showing consistent numerical and spatial distributions between the modified RSEI and oRSEI [21]. Similarly, Li et al. enhanced the evaluation of the Central Yunnan Urban Agglomeration by introducing a soil erosion index, with negligible differences observed between the modified RSEI and oRSEI [15]. These studies indicate that while modifications may result in localized differences, they do not significantly alter the overall effectiveness of ecological quality evaluation at regional scales. This indicates that the incorporation of supplementary factors may not significantly enhance the precision of ecological quality evaluation.

In this study, we recognized the limitations of the NDVI in accurately characterizing vegetation in drylands and therefore replaced it with the MSAVI to construct the aRSEI. The findings showed that the aRSEI slightly enhanced the ecological quality value in vegetated areas, maintained it in areas with very low to low vegetation cover, and reduced it in bare lands. This substitution underscores the importance of selecting appropriate indices that accurately reflect regional ecological conditions. It highlights the need for careful consideration when modifying the RSEI or introducing new indices to ensure that such changes genuinely enhance the evaluation’s accuracy and reliability across different ecological contexts. Consequently, when conducting ecological quality research at regional or larger scales, it is of paramount importance to select appropriate indices based on regional characteristics and ecological objectives. Furthermore, we recommend that any modifications to the RSEI be undertaken with caution, unless there are clear advantages to be gained, in order to avoid introducing unnecessary uncertainties.

### 3.2. Spatio-Temporal Heterogeneity and Driving Force of Ecological Quality Change

The spatial and temporal changes in ecological quality in the study area were examined from 2000 to 2022, revealing clear heterogeneous patterns and gradient characteristics. These findings provide a scientific basis for regional ecological environment monitoring and zoning management. The results of multiple regression residual analysis (Figure 7 and Table 5) indicate that changes in ecological quality were primarily driven by both climate and human factors, with climate change mainly responsible for quality decline and human activities predominantly influencing improvements.

Throughout the study period, we observed increasing trends in precipitation, temperature, and potential evapotranspiration (Figure 8). These climatic changes have complex and often contradictory effects on dryland ecosystems. While increased rainfall can promote vegetation growth and enhance soil moisture retention, higher temperatures and elevated evapotranspiration rates can lead to water stress, reduced plant productivity, and potential soil degradation [30]. Consequently, these interacting factors result in diverse ecological outcomes across different parts of the study area, highlighting the complex nature of climate-driven changes in dryland ecosystems. This dynamic is particularly evident in the desert and Gobi areas outside the Yellow River, where the poor ecological background amplifies the negative effects of increasing temperature and potential evapotranspiration, outweighing the positive impacts of precipitation. Moreover, fewer ecological projects such as grass–animal balance and grassland restoration were implemented in these areas. Conversely, the inner areas of the Yellow River experiences relatively weaker negative effects from climate change due to higher precipitation and better vegetation cover and also have benefited from numerous ecological projects. These initiatives have led to continuous improvements in vegetation conditions and significant ecological restoration of the Kubuqi Desert, Mu Us Sandy Land, and Ordos Steppe [31,32,33].

The factors influencing changes in ecological quality change over time. From 2000 to 2011, human activities contributed significantly to both improvement (68.31%) and decline (83.36%) in ecological quality. The inner areas of the Yellow River benefited from favorable hydrothermal conditions, the successive implementation of ecological projects, and the rapid expansion of cropland. During this period, barren mountains and sandy lands gradually turned green, and desertification control has achieved remarkable results. In contrast, the outside areas suffered from a fragile ecological environment exacerbated by overgrazing and mining activities. The period from 2012 to 2022 witnessed a gradual shift in the balance of influencing factors, with the role of human activities diminishing and climate factors gaining prominence. The areas suitable for ecological projects in the inner regions greatly decreased, and the process of oasis formation slowed [34]. Simultaneously, the implementation of forage–livestock balance and pasture restoration projects in outside areas helped mitigate the negative impacts of grazing and other human activities.

Such factors were the primary drivers of ecological quality changes and transitions between different quality levels. Notably, high-quality ecological areas continued to decrease during the study period, potentially due to changes in cropping patterns. The Ningxia and Hetao Plains, once characterized by the extensive cultivation of water-intensive crops such as rice and wheat, have undergone a significant shift in agricultural practices. The gradual reduction in water-intensive crop cultivation, coupled with the implementation of more efficient water utilization methods and water-saving irrigation techniques like drip irrigation, led to decreased regional humidity. Paradoxically, these water conservation efforts, while beneficial for sustainable water management, contributed to a decline in ecological quality in these areas, highlighting the complex relationship between agricultural practices, water use efficiency, and ecosystem health in drylands.

Furthermore, the expansion of urban and industrial land, encroaching upon cropland and the steppe, had a significant negative effect on ecological quality. Our findings indicate a decrease in ecological quality across all urban areas in the study area except for the urban area of Ordos [35,36]. Ordos’s unique situation may be attributed to its relatively recent development and the higher proportion of forest and grass vegetation in the urban area. These variations in ecological quality among cities offer valuable insights for future urban planning and development strategies, highlighting the need for balanced approaches that consider both economic growth and ecological preservation.

### 3.3. Strategies for Ecological Protection and Restoration

The Hurst exponent persistence analysis has identified areas that are projected to experience continued decline or transition from improvement to decline in the future. These areas should be designated as critical ecological zones, requiring targeted protection and restoration measures. This proactive approach is essential for preventing further degradation and maintaining overall ecological stability in the region.

Areas prone to sustained ecological decline are predominantly located west and north of the Yellow River. While ecological quality changes in these areas are primarily driven by climatic factors and face significant challenges, our research indicates that proactive measures can effectively mitigate adverse effects [36]. To address these challenges, we recommend expanding the scope and intensity of returning grazing land to grass restoration initiatives. This approach should be coupled with completely transforming the forage–livestock balance into a strict grazing prohibition. Additionally, the pruning of dryland shrubs to promote plant growth in areas with relatively good hydrothermal conditions is advised.

For areas of future decline, it is crucial to build upon existing ecological protection and restoration achievements. These areas generally have a better ecological foundation, and future efforts should focus on preventing damage from human activities. This can be achieved by strictly implementing the system of ecological protection red lines and rigorously enforcing policies on grazing prohibition and forage–livestock balance. Moreover, the continuation of ecological restoration projects, such as afforestation and grass planting, is imperative, with due consideration given to the principle of matching appropriate vegetation to suitable locations.

In addition, specific measures should be taken for urban and cropland areas. In urban areas, development should prioritize intensive land use practices and improve ecological quality through rational planning and design. For farmland areas, the optimization of the allocation of protective forests and the intensification of residential area greening efforts are recommended.

To ensure the efficacy of these strategies and provide data for future management decisions, we propose the implementation of long-term field surveys and ecological monitoring. These efforts should focus particularly on areas where ecological quality declined between 2000 and 2022. The findings of this ongoing research will provide a robust scientific basis for refining our approach to ecological protection, restoration, and resource utilization in the study area [37,38].

### 3.4. Limitations and Perspectives

We analyzed ecological quality changes in the study area over the past 23 years, demonstrating the efficacy of the adaptive model modifications. However, several areas for methodological improvement have been identified. Firstly, although the RSEIs are widely used for ecological quality evaluation, their validation is primarily reliant on image visualization. In the future, practical scientific validation methods incorporating field surveys and data monitoring should be developed to ensure the accuracy and reliability of evaluation results. Secondly, the MSAVI was employed as a greenness indicator in drylands, achieving a more favorable application compared to that of the oRSEI. However, it remains to be seen whether a series of vegetation indices, such as the EVI and SAVI, are more suitable than the MSAVI in drylands. Furthermore, while the MODIS data (500 m resolution) used in this study offer advantages for large-scale, continuous time series evaluation, it may not capture fine-scale spatial and temporal variations in ecological quality [39,40]. This limitation suggests potential benefits from integrating higher-resolution data in future studies. Additionally, the multiple regression residual analysis remains inadequate for accurately quantifying the impacts of individual drivers [28]. In the next step, advanced techniques, such as machine learning, can be employed to quantify and refine the relative contributions of different drivers.

By addressing these limitations and pursuing these future directions, we can enhance the understanding of ecological quality dynamics and provide more robust scientific foundations for environmental management and policy decisions in fragile ecosystems.

## 4. Materials and Methods

### 4.1. Study Area

**Figure 9:**
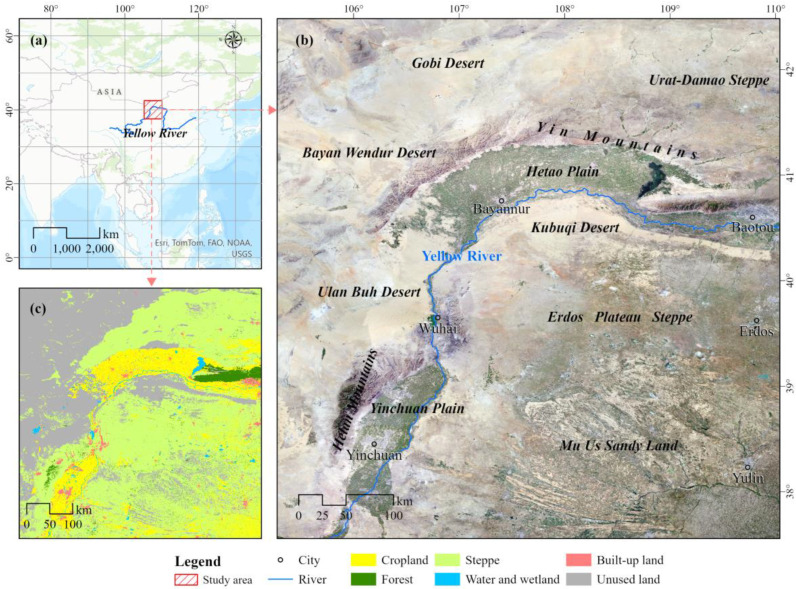
Overview of the study area. (**a**) Location of the study area. (**b**) ArcGIS online map (World Imagery). (**c**) Land use map in 2020 (GlobeLand30).

The study area is situated in northern China (37°33.98′–42°28.67′ N, 105°14.89′–110°25.64′ E). The Yellow River traverses the study area, naturally dividing it into two distinct sections (Figure 9). The outer areas of the river include the Yinchuan Plain, Hetao Plain, Helan Mountains, Yin Mountains, Ulan Buh Desert, Bayan Wendur Desert, Gobi Desert, and Urat-Damao steppe. The inner areas of the river encompass the Kubuqi Desert, Ordos Plateau Steppe, and Mu Us Sandy Land. The study area covers a wide range of climate types including arid, semi-arid, and dry sub-humid ones, and it is dominated by arid and semi-arid climates. Annual precipitation varies from 68 to 477 mm, and the mean annual temperature spans from −1.9 °C to 9.6 °C. The topography features significant elevation differences, with altitudes ranging from 806 m to 3337 m above sea level. This region features high solar radiation and elevated potential evapotranspiration rates [22]. The landscape is characterized by a complex mosaic of ecosystems, including the desert, mountains, the Gobi, and steppe vegetation communities with relatively low biodiversity.

The population distribution in the study area is characterized by a concentration in the plains, where the majority of economic activities takes place. Agriculture represents the primary economic activity in the region, with sunflower and corn being the primary crops cultivated. Animal husbandry, particularly sheep farming, represents another significant economic activity in the region. While the agricultural sector is the dominant force, the industrial sector is also present but relatively underdeveloped. Industrial activities are mainly limited to resource extraction and primary processing, which reflects the region’s early stage of industrial development.

### 4.2. Data and Processing

#### 4.2.1. Satellite Data

MODIS data were chosen for their high-frequency observations and diverse data products which are essential for capturing dynamic environmental and ecological changes [41]. The NDVI was derived from the MOD13A1 16-day composite product with 500 m spatial resolution. The LST was derived from the MOD11A2 1 km resolution surface temperature/emissivity 8-day composite product and resampled to 500 m resolution for consistency. The MSAVI, the NDBSI, WET, the ST, and the MNDWI were calculated from the MOD09A1 500 m resolution surface reflectivity 8-day composite product. MODIS images from 1 April to 30 September, which correspond to the vegetation growing season, were selected for the years 2000 to 2022 using the GEE. These images were then processed with a median reducer, which selected the median pixel value from the image stack to minimize atmospheric interference and cloud contamination [42].

#### 4.2.2. Climate Data

The annual precipitation, annual mean temperature, and potential evapotranspiration data, with 1 km spatial resolution, were obtained from the National Tibetan Plateau/Third Pole Environment Data Center (http://data.tpdc.ac.cn) [43,44,45,46,47]. These data were uniformly resampled to 500 m resolution to ensure consistency with the MODIS data.

### 4.3. Technical Workflow

**Figure 10:**
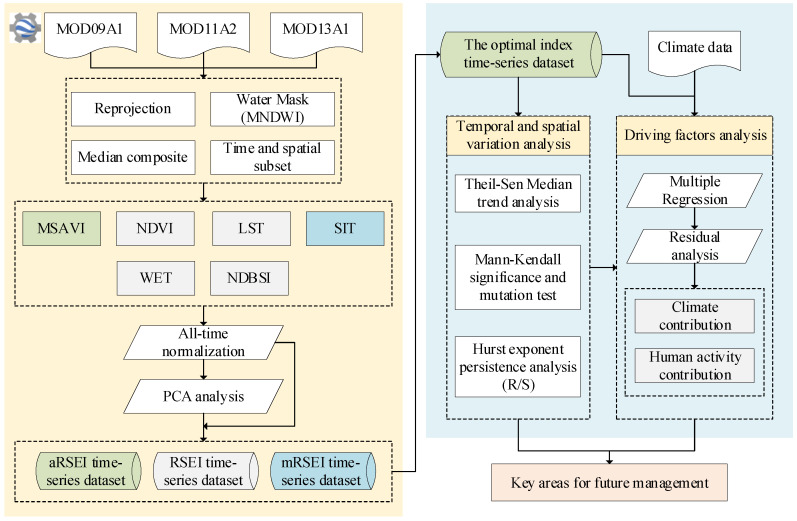
Technical workflow.

In this study, the Google Earth Engine (GEE) platform was used to analysis the spatial and temporal changes in ecological quality in a typical dryland region. The technical workflow is depicted in Figure 10. The main process encompasses several key steps. First, MODIS data underwent pre-processing, followed by calculation and global normalization of ecological indicators. RSEIs were then constructed, and the optimal index was identified. Spatio-temporal changes in ecological quality were analyzed using Theil–Sen median trend analysis, and Mann–Kendall significance and mutation tests. The persistence of ecological quality changes was further assessed using the Hurst exponent. Finally, multiple residual regression analysis was applied to quantitatively evaluate the driving forces behind these changes.

### 4.4. RSEIs Construction

#### 4.4.1. Original RSEI

The most widely used oRSEI, developed by Xu et al. [7], represents the regional ecological environment using four components: greenness, humidity, dryness, and heat. Principal component analysis (PCA) was performed to combine these ecological indicators, and the first component of PCA (PC1) is selected to represent ecological quality. The equation is shown as follows:

(1) oRSEI=PC1(Greenness,Humidity,Heat,Dryness)

oRSEI refers to the RSEI without modifications. Greenness is characterized by the NDVI. Humidity was calculated by the wet component of the Tasseled Cap Transformation. Heat is represented by the LST. Dryness uses the NDBSI, which is calculated using the Index of Building (IBI) and the Soil Index (SI). The ecological indicators were obtained and calculated using the formulae shown in Table 6.

#### 4.4.2. Modified RSEI

In drylands, pervasive soil salinization causes a decline in soil quality, leading to regional ecological degradation. Therefore, soil salinization is often considered an important ecological indicator affecting regional ecological quality and is added to the oRSEI to construct new RSEIs [14,20,21]. Accordingly, we constructed a modified RSEI (mRSEI) by adding a salinity index (ST, the formula is shown in Table 6) to the oRSEI. The equation is shown as follows:

(2) mRSEI=PC1(NDVI,LST,WET,NDBSI,ST)

#### 4.4.3. Adapted RSEI

Due to the limitations of the NDVI in low vegetation cover areas, where it is easily affected by soil background, and in high vegetation cover areas, where it exhibits saturation effects [48,49], it is challenging to accurately reflect vegetation changes in both low and high vegetation cover areas. In contrast, the MSAVI [50], by introducing a soil adjustment factor, reduces interference from the soil background, reflecting vegetation conditions more accurately in high cover areas and being more sensitive to vegetation changes in low cover areas. Considering the limitations of the NDVI in representing vegetation in drylands and the advantages of the MSAVI in such regions, we also constructed an aRSEI by replacing the NDVI with the more suitable MSAVI (the formula is shown in Table 6). The equation is shown as follows:

(3) aRSEI=PC1(MSAVI,WET,LST,NDBSI)

**Table 6:** Calculation of ecological indicators.

Factors	Indicators	Formula	Description
Greenness	NDVI	MOD13A1	The NDVI and MSAVI can intuitively reflect the health status of surface vegetation, and both of them are widely used in vegetation dynamics monitoring.
	MSAVI	$\begin{array}{l} MSAVI\\ =\left(2\times nir+1-\sqrt{{\left(2\times nir\right)}^{2}-8\times \left(nir-red\right)}\right)/2 \end{array}$	
Humidity	WET	$\begin{array}{c} WET=0.1147B1+0.2489B2+0.2408B3+0.3132B4\\ -0.3122B5-0.6416B6-0.5087B7 \end{array}$	WET can better reflect the surface vegetation and soil moisture status, and it is also closely related to ecological changes such as soil degradation.
Dryness	NDBSI	NDBSI=(SI+IBI)/2 $SI=\frac{\left(B6+B1)-(B2+B3\right)}{\left(B6+B1)+(B2+B3\right)}$ $IBI=\frac{\frac{2\cdot B6}{B6+B2}-\frac{B2}{B2+B1}+\frac{B4}{B4+B6}}{\frac{2\cdot B6}{B6+B2}+\frac{B2}{B2+B1}+\frac{B4}{B4+B6}}$	The NDBSI can be used to characterize the drying status of the surface [42,51].
Heat	LST	MOD11A2	LST directly affects plant growth, soil moisture, species distribution, and ecosystem functions [52].
Salinity	ST	$ST=\sqrt{red\times blue}$	The ST reflects the salinization status of the region, significantly impacting vegetation distribution and soil quality.

#### 4.4.4. Global Normalization

To avoid the imbalance of weights among ecological indicators caused by the non-uniformity of scale, it is necessary to normalize the indicators. Existing studies generally employ the approach of normalizing them on an annual basis individually [53]. However, this methodology fails to take into account the influence of multiple factors arising from the temporal variations presented by ecological indicators [54]. Therefore, we normalized the indicators using global normalization, which is achieved by obtaining the maximum and minimum values of each indicator in time and space. Global normalization makes the temporal results of each indicator comparable.

(4) $$y_{i}=\left(x_{i}-x_{min}\right)/\left(x_{max}-x_{min}\right)$$

where *y_i_* denotes the global normalized value of indicator *x*, *x_i_* is the original pixel value of *x*, *x_max_* is the global maximum value of *x*, and *x_min_* is the global minimum value of *x*.

Additionally, after performing PCA on the ecological indicators to obtain the PC1, it is also necessary to normalize it to constrain the ecological quality values within the [0–1] range. Normalizing the PC1 for a single year still presents the issue of temporal variation. Therefore, we applied global normalization to ensure that the ecological quality results are suitable for temporal comparisons.

However, it should also be noted that using uniform maximum and minimum values for multiple years in global normalization erases absolute value differences between different years, resulting in the same annual mean after normalization [55]. This makes it impossible to use the mean to characterize changes over the overall time series of the study area. Nonetheless, this method provides more accurate results for class changes and time series changes in ecological quality based on pixels.

### 4.5. Pixel-Level Dynamic Detection

The Theil–Sen median trend analysis and Mann–Kendall test were used to analyze the time series changes in ecological quality [56]. The Theil–Sen median trend analysis is a robust nonparametric statistical method for trend calculation, being computationally efficient, insensitive to measurement errors and outliers, and suitable for trend analysis of long time series data. The calculation formula is as follows:

(5) $$\beta =median\left(\frac{x_{j}-x_{i}}{j-i}\right),\forall j>i$$

where *β* denotes the trend of ecological quality, and median() is the median function. *x_i_* and *x_j_* denote the value of ecological quality in year *i* and year *j.* When *β* > 0, the ecological quality indicates an increasing trend; otherwise, it indicates a decreasing trend.

The Mann–Kendall (M-K) test is a nonparametric test for trends in time series, which does not require the measurements to follow a normal distribution, is unaffected by missing values and outliers [57], and is suitable for trend significance tests and mutation analysis of long time series data. The M-K test is calculated as follows:

(6) $$Z=\left\{\begin{array}{c} \frac{s}{\sqrt{Var(S)}}\left(S>0\right)\\ 0\left(S=0\right)\\ \frac{s+1}{\sqrt{Var(S)}}\left(S<0\right) \end{array}\right.$$

(7) $$S=\sum_{i=1}^{n-1}\sum_{j=i+1}^{n}sgn\left(x_{j}-x_{i}\right)$$

(8) $$\beta sgn\left(x_{j}-x_{i}\right)=\left\{\begin{array}{c} 1\left(x_{j}-x_{i}>0\right)\\ 0\left(x_{j}-x_{i}=0\right)\\ -1\left(x_{j}-x_{i}<0\right) \end{array}\right.$$

(9) $$Var\left(S\right)=\left(n(n-1)(2n+5)\right)/18$$

where *x_i_* and *x_j_* denote the value of *x* in year *i* and year *j*, respectively; *n* denotes the length of the time series; *sgn*() is the sign function; and Z refers to standardized test statistics. |Z| ≥ 1.96 at a 0.05 significance level indicates that the change trend of the time series passes the test of significance with a confidence level of 95%. Combined with the β value in the Theil–Sen median trend, the ecological quality change of pixels was categorized into five categories: significant increase, slight increase, stable, slight decrease, and significant decrease.

The M-K mutation test was performed on the mean ecological quality values in areas of significant change to ascertain the year in which regional ecological quality reached a significant level in comparison to that in 2000. Additionally, the test was employed to investigate the potential occurrence of mutations. The calculation procedure is described in Qi et al. [58].

### 4.6. The Persistence of Change and Future Trends

The Hurst exponent [59] is an important tool for analyzing changes in time series data that reflect long-term dependency [4,60]. This long-term dependency indicates the consistency between future and current change trends. In this study, we used the rescaled range (R/S) analysis method to calculate the Hurst exponent [61,62].

Based on the value of the Hurst exponent (H), it can be determined whether the ecological quality change is persistent. If H ˃ 0.5, the future change trend will be persistent; when H = 0.5, the future change will be random; and when H ˂ 0.5, the future change trend will be opposite to the current trend. Combining the Hurst exponent with the results of Theil–Sen median trend analysis provides a comprehensive understanding of the persistence of future changes in ecological quality.

### 4.7. Multiple Regression Residual Analysis

Multiple regression residual analysis [63] was employed to evaluate the influence and relative contributions of climate change and human activities to ecological quality changes, identifying the dominant drivers of these changes [36]. The impacts of climate change are often studied only in terms of precipitation and temperature, as seen in many studies in drylands [64,65]. However, potential evapotranspiration in drylands is also a key factor affecting regional ecology [66]. In this study, we used ecological quality values as the dependent variable, and annual precipitation, annual mean temperature, and potential evapotranspiration as independent variables to establish a multiple linear regression model and calculate the model parameters. Based on these independent variables and the regression model parameters, we predicted values (RSEIcc) under changing climatic factors, which represent the impact of climate change. Then, the difference between actual ecological quality values (RSEI) and RSEIcc was calculated and defined as the RSEI residual (RSEI_HA_), to represent the impact of human activities. The calculation equations are as follows:

(10) RSEI=a×Pre+b×Tem+c×Pet+d

(11) $${RSEI}_{HA}=RSEI-{RSEI}_{CC}$$

where *a*, *b*, *c*, and *d* are the model parameters; *Pre* represents annual precipitation; *Tem* represents annual mean temperature; and *Pet* represents potential evapotranspiration.

The linear trend rates of RSEI_CC_ and RSEI_HA_ represent the changes in the RSEI under the influence of climate change and human activities, respectively [35,67]. A positive trend rate indicates that climate change or human activities improve the RSEI, while a negative trend rate indicates they decrease the RSEI. Consequently, we can calculate the relative contributions of the drivers of changes in ecological quality (Table 7). It is worth noting that the residual analysis method has some limitations and uncertainties [68,69], especially in areas with slight changes, which may be influenced by various factors, such as the quality of remote sensing imagery and surface conditions. Therefore, this study quantitatively analyzed the driving forces in areas with significant changes in ecological quality.

## 5. Conclusions

This study aimed to assess the regional applicability of three RSEIs (oRSEI, mRSEI, and aRSEI) in a typical dryland in northern China. Furthermore, the spatial and temporal characteristics of ecological quality were evaluated based on the optimal index, quantifying the relative contributions of climate change and human activities as driving factors. The main conclusions are as follows:

(1) Three RSEIs reflected the overall pattern and spatial differences in regional ecological quality. The aRSEI, which used the MSAVI, provided the most balanced evaluation of vegetated and non-vegetated features, outperforming the oRSEI and mRSEI.
(2) The changes in ecological quality from 2000 to 2022 were heterogeneous, with significant improvements observed in the inner areas of the Yellow River and declines in the outer area. The rate of change in ecological quality was found to be greater during the period from 2000 to 2011 than during the subsequent period from 2012 to 2022.
(3) The combined effect of climate change and human activities led to changes in ecological quality in the study area. The analysis revealed that human activities were the primary driver of changes from 2000 to 2011. During this period, human activities contributed to 68.31% of the improvement in ecological quality and 83.36% of the decline. Meanwhile, climate change played a more significant role between 2012 and 2022, contributing 59.67% to improvement and 40.03% to decline.
(4) Human activities can effectively mitigate ecological degradation. For areas facing potential future decline, it is crucial to implement ecological protection and restoration measures to address and alleviate ecological challenges.

This study systematically compares the applicability of different remote sensing ecological indices for the evaluation of ecological quality in drylands. It provides theoretical support and technical reference for regional ecological protection strategies by revealing the spatio-temporal characteristics of ecological quality and its driving mechanisms.

## Figures and Tables

**Table 1:** PCA results of three ecological remote sensing indices in 2022.

Index	Contribution (%)	Eigen Vectors					
		NDVI	WET	LST	NDBSI	MSAVI	SIT
oRSEI	82.11	−0.5667	−0.5370	0.4527	0.4309		
mRSEI	82.38	−0.4895	−0.4714	0.3878	0.3574		0.5100
aRSEI	82.38		−0.5429	0.4612	0.4296	−0.5550	

**Table 2:** Correlation between ecological quality indices and ecological indicators in 2022.

Correlation	NDVI	MSAVI	WET	LST	NDBSI	SIT	Mean
oRSEI	0.9337	0.9302	0.9019	−0.8592	−0.9239	−0.8808	0.9049
mRSEI	0.9294	0.9378	0.9124	−0.8481	−0.8830	−0.9361	0.9078
aRSEI	0.9195	0.9353	0.9053	−0.8691	−0.9145	−0.8918	0.9059

**Table 3:** Ecological quality change trends from 2000 to 2020.

β (aRSEI)	|Z|	Trends	Percentage (%)
˃0	≥1.96	Significant improvement	16.77
	˂1.96	Slight improvement	13.96
˂0	≥1.96	Significant decline	18.09
	˂1.96	Slight decline	28.35
=0	-	No change	22.83

**Table 4:** Ecological quality change trends from 2000 to 2020.

Hurst	β (aRSEI)	Persistence of Future Trends	Percentage (%)
˃0.5	˃0	Continuous improvement	8.75
	˂0	Continuous decline	11.76
	=0	Stable	21.89
˂0.5	˃0	Improvement to decline	22.08
	˂0	Decline to improvement	34.87
=0.5	-	Uncertain	0.65

**Table 5:** Driving contributions of climate change and human activities to ecological quality change in the significant-change areas from 2000 to 2022.

Areas	Periods	Climate Change (%)					Human Activities (%)				
		0–25	25–50	50–75	75–100	100	0–25	25–50	50–75	75–100	100
Significant-improvement area	2000–2022	12.84	47.58	31.72	7.17	0.69	7.84	31.69	47.61	12.33	0.53
	2000–2011	50.11	27.07	10.45	4.30	8.07	12.37	10.44	27.06	30.00	20.13
	2012–2022	19.57	15.93	25.50	19.90	19.10	38.98	25.52	15.93	8.84	10.74
Significant-decline area	2000–2022	8.17	33.70	46.98	10.47	0.68	11.13	46.95	33.72	7.32	0.87
	2000–2011	77.16	10.52	4.18	2.02	6.12	8.13	4.18	10.51	24.24	52.94
	2012–2022	31.09	20.06	20.04	11.62	17.18	28.78	20.05	20.07	13.35	17.75

**Table 7:** The relative contributions of climate change and human activities to ecological quality change.

*S_RSEI_*	*S_cc_*	*S_HA_*	Driver	Climate Contribution (%)	Human Contribution (%)	Description
˃0	˃0	˃0	CC&HA	$S_{CC}/S_{RSEI}$	$S_{HA}/S_{RSEI}$	Combined contribution
	˃0	˂0	CC	100	0	Climate dominated increase
	˂0	˃0	HA	0	100	Human activities dominated increase
˂0	˃0	˃0	CC&HA	$S_{CC}/S_{RSEI}$	$S_{HA}/S_{RSEI}$	Combined contribution
	˃0	˂0	CC	100	0	Climate dominated decrease
	˂0	˃0	HA	0	100	Human activities dominated decrease

## Data Availability

The original contributions presented in this study are included in the article, and further inquiries can be directed to the corresponding author.
